# Prognostic Impact of Different Types of Ventricular Tachyarrhythmias Stratified by Underlying Cardiac Disease

**DOI:** 10.3390/jpm12122023

**Published:** 2022-12-07

**Authors:** Tobias Schupp, Jonas Rusnak, Kathrin Weidner, Thomas Bertsch, Kambis Mashayekhi, Péter Tajti, Ibrahim Akin, Michael Behnes

**Affiliations:** 1 Department of Cardiology, Angiology, Haemostaseology and Medical Intensive Care, University Medical Centre Mannheim, Medical Faculty Mannheim, Heidelberg University, 68167 Mannheim, Germany; 2 European Center for AngioScience (ECAS) and German Center for Cardiovascular Research (DZHK) Partner Site Heidelberg/Mannheim, 68167 Mannheim, Germany; 3 Institute of Clinical Chemistry, Laboratory Medicine and Transfusion Medicine, Nuremberg General Hospital, Paracelsus Medical University, 90419 Nuremberg, Germany; 4 Department of Internal Medicine and Cardiology, Mediclin Heart Centre Lahr, 77933 Lahr, Germany; 5 Gottsegen György National Cardiovascular Center, 1096 Budapest, Hungary

**Keywords:** sudden cardiac death, ventricular tachycardia, ventricular fibrillation, mortality, ventricular tachyarrhythmia

## Abstract

Limited data regarding the outcome of patients with different types of ventricular tachyarrhythmias is available. This study sought to assess the prognostic impact of different types of ventricular tachyarrhythmias stratified by underlying cardiac disease. A large retrospective registry was used including all consecutive patients presenting with ventricular tachycardia (VT) and fibrillation (VF) on admission from 2002 to 2016. Patients with non-sustained VT (ns-VT), sustained VT (s-VT) and VF were compared using uni- and multivariable Cox regression models. Risk stratification was performed after stratification by underlying cardiac disease (i.e., acute myocardial infarction (AMI), ischemic heart disease (IHD), non-ischemic cardiomyopathy (NICM) and patients considered as lower-risk for ventricular tachyarrhythmias). The primary endpoint was defined as all-cause mortality at 2.5 years. Secondary endpoints were cardiac death at 24 h, all-cause mortality at 5 years, cardiac rehospitalization and a composite arrhythmic endpoint at 2.5 years. In 2422 consecutive patients with ventricular tachyarrhythmias, most patients were admitted with VF (44%), followed by ns-VT (30%) and s-VT (26%). Patients with VF suffered most commonly from AMI (42%), whereas heart failure was more common in s-VT patients (32%). In patients with AMI (HR = 1.146; 95% CI 0.751–1.750; *p* = 0.527) and in the lower-risk group (HR = 1.357; 95% CI 0.702–2.625; *p* = 0.364), the risk of all-cause mortality did not differ in VF and s-VT patients. In IHD patients, VF was associated with impaired prognosis compared to s-VT (HR = 2.502; 95% CI 1.936–3.235; *p* = 0.001). In conclusion, VF was associated with worse long-term prognosis compared to s-VT in IHD patients, whereas the risk of all-cause mortality among VF and s-VT patients did not differ in patients with AMI, NICM and in patients considered at lower risk for ventricular tachyarrhythmias.

## 1. Introduction

As a consequence of preventive measures and nationwide resuscitation programs in the Western world, cardiovascular mortality has decreased within the last decades [1]. About 17 million deaths per year are still caused by cardiovascular diseases and approximately 25% are related to sudden cardiac death (SCD) [1,2,3,4]. Underlying pathologies for SCD are manifold, including most frequently coronary artery disease (CAD) and acute myocardial infarction (AMI), followed by non-ischemic cardiomyopathies (NICM) and rare causes, including electrolyte disorders, subarachnoidal haemorrhage, pulmonary embolism and idiopathic ventricular tachyarrhythmias [2,5,6]. Although heart failure with severely reduced left ventricular ejection fraction (LVEF) was demonstrated to be an important predictor for ventricular tachyarrhythmias, most SCD cases occur in patients with no evidence of heart failure or structural heart disease [7].

The pathophysiology of ventricular tachyarrhythmias is complex; however, three major mechanisms have been identified: re-entry, enhanced automaticity and triggered activity. Re-entry represents the most common etiology of ventricular tachyarrhythmias in patients with structural heart disease and occurs when the action potential reactivates a region that has recovered excitability [8]. Automaticity is the consequence of spontaneous depolarization of cardiac cells in the absence of external electrical stimulation and may occur as a result of ischemia or AMI [9]. Triggered activity may be caused by afterdepolarizations, which are depolarizing oscillations in membrane voltage as a result of preceding action potentials [10]. The occurrence of ventricular tachyarrhythmias was demonstrated to be associated with increased risk of all-cause mortality in patients with AMI, heart failure, NICM and cardiogenic shock; however, no further risk stratification according to the type of underlying ventricular arrhythmia was performed [11,12,13].

Although ventricular tachyarrhythmias are associated with poor clinical outcomes, direct comparisons of different types of ventricular tachyarrhythmias are limited at a time of high implantable cardioverter-defibrillator (ICD) supply, as well as being stratified by different cardiac diseases. Therefore, this study aims to evaluate the characteristics and underling cardiac pathologies in patients with different types of ventricular tachyarrhythmias (i.e., ventricular fibrillation (VF), sustained ventricular tachycardia (s-VT) or non-sustained VT (ns-VT)), as well as the prognostic role of different types of ventricular tachyarrhythmias stratified by underlying cardiac disease.

## 2. Materials and Methods

### 2.1. Study Patients, Design and Data Collection

The present study included all patients presenting with ventricular tachyarrhythmias from 2002 until 2016 at one institution. Using the electronic hospital information system, all relevant clinical data related to the index event were documented. Further data being documented contained baseline characteristics, prior medical history, prior medical treatment, length of index stay, detailed findings of laboratory values at baseline, data derived from all non-invasive or invasive cardiac diagnostics and device therapies, such as coronary angiography, electrophysiological examination, and data being derived from prior or newly implanted cardiac devices, including already implanted at index and ICD at follow-up. Every re-visit at the out-patient clinic or leading to rehospitalization was documented when related to recurrent ventricular tachyarrhythmias and adverse cardiac events. Documentation period lasted from index event until 2016. Documentation of all medical data was performed by independent cardiologists at the time of the patients’ individual period of clinical presentation, being blinded to final data analyses.

The present study is derived from an analysis of the “Registry of Malignant Arrhythmias and Sudden Cardiac Death—Influence of Diagnostics and Interventions (RACE-IT)” and represents a single-center registry including consecutive patients presenting with ventricular tachyarrhythmias and aborted cardiac arrest being acutely admitted to the University Medical Center Mannheim (UMM), Germany (clinicaltrials.gov identifier: NCT02982473) from 2002 until 2016. The registry was carried out according to the principles of the Declaration of Helsinki and was approved by the Medical Ethics Committee II of the Medical Faculty Mannheim, University of Heidelberg, Germany.

The medical center includes a general emergency department (ED) for emergency admission of traumatic, surgical, neurological and cardiovascular conditions. Interdisciplinary consultation is an inbuilt feature of this 24/7 service, and connects to a stroke unit, four intensive care units (ICU) with extracorporeal life support, and a chest pain unit (CPU) to alleviate rapid triage of patients. The cardiologic department itself includes a 24 h catheterization laboratory, an electrophysiologic laboratory, a hybrid operating room and telemetry units.

### 2.2. Definition of Study Groups, Inclusion and Exclusion Criteria

For the present analysis, patients presenting with ventricular tachyarrhythmias on admission were included. Ns-VT, s-VT or VF were defined according to current European guidelines [1]. S-VT was defined by duration of more than 30 s or causing hemodynamic collapse within 30 s. Ns-VT was defined by duration of less than 30 s along with wide QRS complex (≥120 milliseconds) at a rate greater than 100 beats per minute [1]. Ventricular tachyarrhythmias were documented by 12-lead echocardiogram (ECG), ECG tele- monitoring, ICD or, in case of unstable course or during resuscitation, by external defibrillator monitoring. Documented VF was treated by external defibrillation and, in case of prolonged instability, with additional intravenous anti-arrhythmic drugs during cardiopulmonary resuscitation (CPR). Patients with episodes of VT plus VF were included in the VF group, whereas patients with ns-VT plus s-VT were included in the s-VT group.

Overall exclusion criteria comprised patients without complete follow-up data regarding mortality. Each patient was counted only once for inclusion when presenting with the first episode of ventricular tachyarrhythmias. Patients without documented ventricular tachyarrhythmias were excluded.

Risk stratification for the comparisons of ns-VT, s-VT and VF was performed according to the presence of AMI, IHD, NICM, and for patients considered at “lower risk”. AMI was defined as previously described according to current international guidelines [14]. ST-segment myocardial infarction (STEMI) was defined as a novel rise in the ST segment in at least two contiguous leads with ST-segment elevation ≥ 2.5 mm in men < 40 years, ≥2 mm in men ≥ 40 years, or ≥1.5 mm in women in leads V2–V3 and/or 1 mm in the other leads. Non-ST-segment myocardial infarction (NSTEMI) was defined as the presence of an acute coronary syndrome with a troponin I increase of above the 99th percentile of a healthy reference population in the absence of ST segment elevation, but persistent or transient ST segment depression, inversion or alteration of T wave, or normal ECG, in the presence of a coronary culprit lesion. 

IHD group comprised patients with evidence of at least one coronary artery stenosis of at least 50% in at least one major epicardial coronary artery, or prior percutaneous coronary intervention (PCI) irrespective of the degree of LVEF in the absence of AMI [15].

The NICM group comprised patients with LVEF < 55% in the absence of CAD, valvular and congenital heart disease sufficient to cause the observed myocardial abnormality. The NICM group comprised dilated cardiomyopathy, hypertrophic (non-)obstructive cardiomyopathy, arrhythmogenic right ventricular dysplasia and non-compaction cardiomyopathy.

Finally, patients with none of the mentioned underlying cardiac disease, no evidence of channelopathy and preserved LVEF (i.e., LVEF ≥ 55%) were included in the lower-risk group.

### 2.3. Study Endpoints

The primary endpoint was all-cause mortality at 2.5 years of follow-up according to the median follow-up period of the study population. Secondary endpoints comprised cardiac death at 24 h, all-cause mortality at 5 years, cardiac rehospitalization at 2.5 years and a composite arrhythmic endpoint (defined as recurrent ventricular tachyarrhythmias or appropriate ICD therapy) at 2.5 years of follow-up.

For the evaluation of the primary endpoint all-cause mortality and the secondary endpoint cardiac death at 24 h and all-cause mortality at 5 years, all patients were included, whereas only patients surviving the index event and being discharged alive were included for the evaluation of the secondary endpoints of cardiac rehospitalization and the composite arrhythmic endpoint.

Overall follow-up period lasted until 2016. All-cause mortality was documented using our electronic hospital information system and by directly contacting state resident registration offices (“Bureau of Mortality Statistics”) across Germany. Identification of patients was verified by name, surname, day of birth and registered living address. Lost to follow-up rate was 1.7% (*n* = 48) regarding survival until the end of the follow-up period.

Cardiac rehospitalization comprised rehospitalization due to recurrent VT or VF, AMI, acute heart failure, CPR and inappropriate ICD therapies. Recurrences of ventricular tachyarrhythmias and appropriate ICD therapies for the evaluation of the composite endpoint were documented reviewing ICD protocols. Device recordings were re-evaluated retrospectively by independent cardiologists being blinded to final data analysis. Appropriate ICD therapies included anti-tachycardia pacing (ATP), ICD-related shock or both ATP and shock in the presence of documented ventricular tachyarrhythmias.

### 2.4. Statistical Methods

Quantitative data are presented as mean ± standard error of mean (SEM), median and interquartile range (IQR), and ranges depending on the distribution of the data, and were compared using the Student’s *t* test for normally distributed data or the Mann-Whitney *U* test for nonparametric data. Deviations from a Gaussian distribution were tested by the Kolmogorov–Smirnov test. Spearman’s rank correlation for nonparametric data was used to test univariate correlations. Qualitative data are presented as absolute and relative frequencies and compared using the Chi^2^ test or the Fisher’s exact test, as appropriate.

Uni- and multivariable Cox regression models were applied for the evaluation of the primary and secondary endpoints in patients with AMI, IHD, NICM and within the lower risk group. Multivariable Cox regression models were performed for the endpoints all-cause mortality at 2.5 years and 5 years, cardiac rehospitalization and the composite endpoint. Logistic regression models were performed for the endpoint cardiac death at 24 h. Multivariable models were applied using the “foreward selection” and adjusted for the following covariables: age, sex, diabetes mellitus, chronic kidney disease (glomerular filtration rate < 60 mL/min/1.73 m^2^), LVEF < 35%, chronic obstructive pulmonary disease (COPD), the presence of a prior AMI, coronary artery bypass grafting (CABG) and the presence of a coronary chronic total occlusion (CTO), that were demonstrated to increase the risk of long-term all-cause mortality within the present study population [16,17,18,19,20,21].

The result of a statistical test was considered significant for *p* < 0.05. SAS, release 9.4 (SAS Institute Inc., Cary, NC, USA) and SPSS (Version 25, IBM, Armonk, NY, USA) were used for statistics.

## 3. Results

### 3.1. Study Cohort

**Figure 1:**
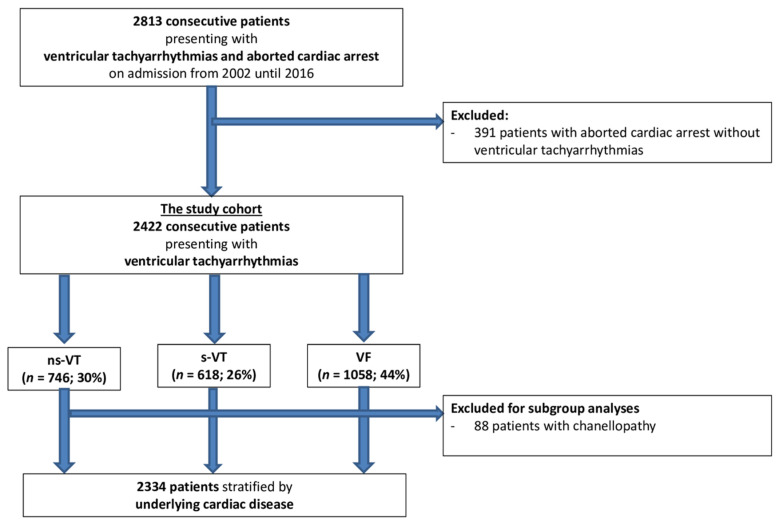
Study population.

This real-life cohort comprised a total of 2422 consecutive patients admitted with ventricular tachyarrhythmias. Most patients had episodes of VF (44%), followed by ns-VT (30%) and s-VT (26%) (Figure 1; Flow chart).

As illustrated in Table 1, patients were median-aged at 68 years. Patients with VF were less likely to be males compared to s-VT patients (70% vs. 75%; *p* = 0.038). Prior heart failure (32% vs. 25% vs. 16%; *p* ≤ 0.003), coronary artery disease (50% vs. 32% vs. 40%; *p* = 0.001), and prior AMI (33% vs. 17% vs. 22%; *p* = 0.001) were mostly seen in patients with s-VT as compared to VF or ns-VT. Furthermore, LVEF < 35% was most common in the s-VT group (48% vs. 30% vs. 34%; *p* = 0.001). Electrophysiological examination, including highest ablation rates, were observed in patients with s-VT as well. An ICD was mostly implanted in patients with s-VT (67%) compared to ns-VT (39%) and VF (47%) (*p* = 0.001). In contrast, AMI on admission was most common in the VF group, followed by ns-VT and s-VT (42% vs. 21% vs. 15%; *p* ≤ 0.003), along with the highest need for CPR (87% vs. 26% vs. 8%; *p* = 0.001). Finally, coronary angiography was more commonly performed in patients with index episodes of VF compared to s-VT (64% vs. 50%; *p* = 0.001), along with higher rates of PCI (60% vs. 28%; *p* = 0.001).

**Figure 2:**
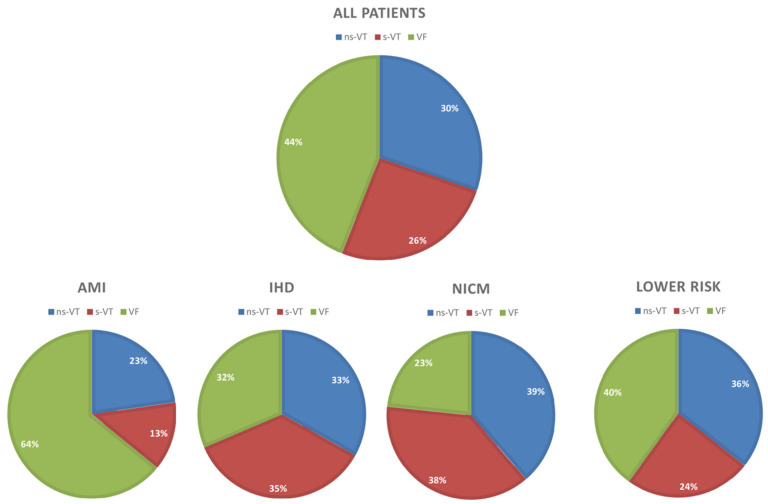
Distribution of different types of ventricular tachyarrhythmias within the entire study cohort, as well as separated by AMI, IHD, NICM and in patients considered at lower risk for ventricular tachyarrhythmias.

The distribution of the types of initial ventricular tachyarrhythmias stratified by the underlying cardiac disease is outlined in Figure 2. Thus, VF was most common in patients presenting with AMI (64%), whereas the rates of s-VT were highest in the IHD group (35%). Ns-VT was most common in patients with NICMP (39%).

### 3.2. Subgroup of AMI Patients

**Figure 3:**
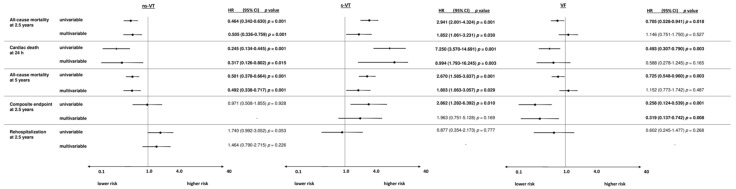
Forest plots demonstrating the uni- and multivariable hazard ratios (HR) with 95% confidence intervals (CI) for the primary endpoint all-cause mortality at 2.5 years, as well as for secondary endpoints all-cause mortality at 24 h, all-cause mortality at 5 years, the composite arrhythmic endpoint (i.e., recurrent ventricular tachyarrhythmias, appropriate ICD therapies) at 2.5 years and cardiac rehospitalization at 2.5 years in AMI patients. Multivariable models were adjusted for age, sex, diabetes, chronic kidney disease, LVEF < 35%, COPD, the presence of a prior AMI, CABG and the presence of a CTO. HRs with corresponding 95% CI were calculated for the comparisons of patients with VF compared to s-VT, s-VT versus ns-VT and ns-VT versus s-VT and VF.

From a total of 691 patients with AMI, most patients had episodes of VF (64%), followed by ns-VT (35%) and s-VT (13%). At 2.5 years, the primary endpoint all-cause mortality occurred in 64% of patients with s-VT, 51% in VF and 31% of patients admitted with ns-VT. Accordingly, the risk of all-cause mortality at 2.5 years was increased in the s-VT group compared to ns-VT patients within the univariable Cox regression analysis (HR = 2.941; 95% CI 2.001–4.324; *p* = 0.001) and lower in the VF compared to the s-VT group (HR = 0.705; 95% CI 0.528–0.941; *p* = 0.001) (Figure 3). In line with this, s-VT was associated with increased risk of cardiac death at 24 h (VF vs. s-VT: HR = 0.493; 95% CI 0.307–0.790; *p* = 0.003; s-VT vs. ns-VT: HR = 7.250; 95% CI 3.570–14.691; *p* = 0.001) and all-cause mortality at 5 years (VF vs. s-VT: HR = 0.493; 95% CI 0.307–0.790; *p* = 0.003; s-VT vs. ns-VT: HR = 7.250; 95% CI 3.570–14.691; *p* = 0.001). In contrast, the risk of the composite arrhythmic endpoint was highest in patients with index episodes of s-VT compared to ns-VT (HR = 2.862; 95% CI 1.282–6.392; *p* = 0.010) and lower in VF compared to s-VT (HR = 0.258; 95% CI 0.124–0.539; *p* = 0.001). Finally, the type of index arrhythmia was not associated with the risk of cardiac rehospitalization.

After multivariable adjustment, the risk of all-cause mortality at 2.5 years (HR = 1.146; 95% CI 0.751–1.750; *p* = 0.527), 24 h (OR = 0.588; 95% CI 0.278–1.245; *p* = 0.165) and 5 years (HR = 1.152; 95% CI 0.733–1.716; *p* = 0.487) did not differ among patients with index episodes of VF compared to s-VT, but was higher in s-VT compared to ns-VT patients (2.5 years: HR = 1.852; 95% CI 1.061–3.231; *p* = 0.030; cardiac death at 24 h: OR = 8.994; 95% CI 1.793–16.245; *p* = 0.003; 5 years: HR = 1.803; 95% CI 1.063–3.057; *p* = 0.029) (Figure 3). After multivariable adjustment, the risk of the composite endpoint was still lower in VF compared to s-VT patients admitted with AMI (HR = 0.319; 95% CI 0.137–0.742; *p* = 0.008). Finally, the risk of cardiac rehospitalization was not affected by the type of index arrhythmia after multivariable adjustment.

### 3.3. Subgroup of Patients with IHD

**Figure 4:**
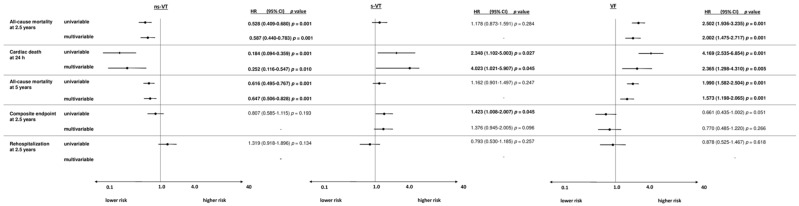
Forest plots demonstrating the uni- and multivariable hazard ratios (HR) with 95% confidence intervals (CI) for the primary endpoint all-cause mortality at 2.5 years, as well as for secondary endpoints all-cause mortality at 24 h, all-cause mortality at 5 years, the composite arrhythmic endpoint (i.e., recurrent ventricular tachyarrhythmias, appropriate ICD therapies) at 2.5 years and cardiac rehospitalization at 2.5 years in IHD patients. Multivariable models were adjusted for age, sex, diabetes, chronic kidney disease, LVEF < 35%, COPD, the presence of a prior AMI, CABG and the presence of a CTO. HRs with corresponding 95% CI were calculated for the comparisons of patients with VF compared to s-VT, s-VT versus ns-VT and ns-VT versus s-VT and VF.

Within the IHD group, most patients were admitted with episodes of s-VT (35%), followed by ns-VT (33%) and s-VT (32%). At 2.5 years, the primary endpoint all-cause mortality occurred in 58% in patients with VF, 31% with s-VT and 28% with ns-VT. Accordingly, the risk of all-cause mortality at 2.5 years (HR = 2.502; 95% CI 1.936–3.235; *p* = 0.001), 24 h (OR = 4.169; 95% CI 2.535–6.854; *p* = 0.001) and 5 years (HR = 1.990; 95% CI 1.582–2.504; *p* = 0.001) was increased in VF compared to s-VT patients within univariable risk prediction models (Figure 4). On the other hand, the risk of all-cause mortality at 2.5 years (HR = 1.178; 95% CI 0.873–1.591; *p* = 0.284) and 5 years (HR = 1.162; 95% CI 0.901–1.497; *p* = 0.247) did not differ among s-VT and ns-VT patients, whereas the risk of cardiac death at 24 h was increased in s-VT patients (OR = 2.348; 1.102–5.003; *p* = 0.027). Furthermore, the composite arrhythmic endpoint occurred more often in s-VT versus ns-VT patients (HR = 1.423; 95% CI 1.008–2.007; *p* = 0.045). In contrast, the rates of cardiac rehospitalization did not differ in patients with different types of ventricular tachyarrhythmias.

After multivariable adjustment, the risk of all-cause mortality at 2.5 years (HR = 2.002; 95% CI 1.475–2.717; *p* = 0.001), cardiac death at 24 h (OR = 2.365; 95% CI 1.298–4.310; *p* = 0.005) and all-cause mortality at 5 years (HR = 1.573 (1.198–2.065; *p* = 0.001) was still increased in VF compared to s-VT patients. In line with this, the risk of cardiac death at 24 h was still higher in s-VT compared to ns-VT (OR = 4.023; 95% CI 1.021–5.907; *p* = 0.045), whereas long-term mortality at 2.5 years (HR = 1.193; 95% CI 0.851–1.674; *p* = 0.306) and 5 years (HR = 1.244; 95% CI 0.937–1.653; *p* = 0.131) did not differ among patients with s-VT or ns-VT (Figure 4). Furthermore, the risk of the composite arrhythmic endpoint did not differ within patients with VF, s-VT or ns-VT. However, patients with ns-VT were associated with the highest risk of cardiac rehospitalization at 2.5 years (HR = 1.524; 95% CI 1.038–2.237; *p* = 0.031).

### 3.4. Subgroup of NICM

**Figure 5:**
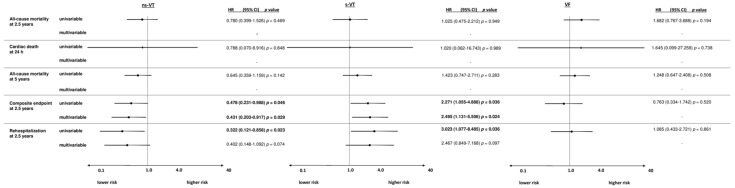
Forest plots demonstrating the uni- and multivariable hazard ratios (HR) with 95% confidence intervals (CI) for the primary endpoint all-cause mortality at 2.5 years, as well as for secondary endpoints all-cause mortality at 24 h, all-cause mortality at 5 years, the composite arrhythmic endpoint (i.e., recurrent ventricular tachyarrhythmias, appropriate ICD therapies) at 2.5 years and cardiac rehospitalization at 2.5 years in NICM patients. Multivariable models were adjusted for age, sex, diabetes, chronic kidney disease, LVEF < 35% and COPD. HRs with corresponding 95% CI were calculated for the comparisons of patients with VF compared to s-VT, s-VT versus ns-VT and ns-VT versus s-VT and VF.

In patients with NICM, ns-VT (39%) was the most common type of index arrhythmia, followed by s-VT (38%) and VF (23%) (Figure 2). In patients with NICM, the type of index arrhythmia had no impact on the risk of all-cause mortality at 2.5 years (VF vs. s-VT: HR = 1.682; 95% CI 0.767–3.688; *p* = 0.194; s-VT vs. ns-VT: HR = 1.025; 95% CI 0.475–2.212; *p* = 0.949; ns-VT vs. s-VT/VF: HR = 0.780; 95% CI 0.399–1.526; *p* = 0.469) (Figure 5). In line with this, the risk of cardiac death at 24 h and all-cause mortality at 5 years was not affected by VF, s-VT or ns-VT. In contrast, the composite arrhythmic endpoint occurred more often in s-VT compared to ns-VT patients (HR = 2.271; 95% CI 1.055–4.888; *p* = 0.036), along with a higher risk of cardiac rehospitalization (HR = 3.023; 95% CI 1.077–8.485; *p* = 0.036).

After multivariable adjustment, the risk of the composite endpoint (HR = 2.495; 95% CI 1.131–5.506; *p* = 0.024) was still higher in s-VT compared to ns-VT patients, whereas the risk of cardiac rehospitalization did not reach statistical significance (HR = 2.467; 95% CI 0.849–7.168; *p* = 0.097).

### 3.5. Lower Risk Patients

**Figure 6:**
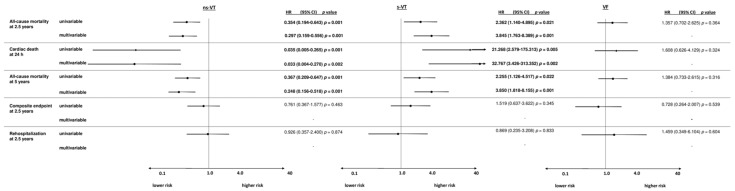
Forest plots demonstrating the uni- and multivariable hazard ratios (HR) with 95% confidence intervals (CI) for the primary endpoint all-cause mortality at 2.5 years, as well as for secondary endpoints all-cause mortality at 24 h, all-cause mortality at 5 years, the composite arrhythmic endpoint (i.e., recurrent ventricular tachyarrhythmias, appropriate ICD therapies) at 2.5 years and cardiac rehospitalization at 2.5 years in patients considered at lower risk. Multivariable models were adjusted for age, sex, diabetes, chronic kidney disease and COPD. HRs with corresponding 95% CI were calculated for the comparisons of patients with VF compared to s-VT, s-VT versus ns-VT and ns-VT versus s-VT and VF.

Finally, patients without AMI, IHD, NICM, channelopathies and preserved LVEF were considered as the lower risk group. In patients considered at lower risk, 40% of the patients were admitted with VF, 36% with ns-VT and 24% with s-VT. The risk of all-cause mortality at 2.5 years (HR = 1.357; 95% CI 0.702–2.625; *p* = 0.364), cardiac death at 24 h (OR = 1.608; 95% CI 0.626–4.129; *p* = 0.324) and all-cause mortality (HR = 1.384; 95% CI 0.733–2.615; *p* = 0.316) was comparable in VF and s-VT patients (Figure 6). In contrast, s-VT patients were associated with increased risk of all-cause mortality at 2.5 years compared to ns-VT (HR = 2.362; 95% CI 1.140–4.895; *p* = 0.021), cardiac death at 24 h (OR = 21.268; 95% CI 2.579–175.313; *p* = 0.005) and all-cause mortality at 5 years (HR = 2.255; 95% CI 1.126–4.517; *p* = 0.022). In contrast, the risk of the composite endpoint and cardiac rehospitalization was not affected by the type of index arrhythmia.

Even after multivariable adjustment, s-VT was associated with increased risk of all-cause mortality at 2.5 years (HR = 3.845; 95% CI 1.763–8.389; *p* = 0.001), cardiac death at 24 h (OR = 32.767; 95% CI 3.426–313.352; *p* = 0.002) and all-cause mortality at 5 years (HR = 3.850; 95% CI 1.818–8.155; *p* = 0.001).

## 4. Discussion

The present study aims to evaluate the characteristics of patients with different types of ventricular tachyarrhythmias (i.e., ns-VT, s-VT or VF) stratified by underlying cardiac diseases including a total of 2422 consecutive patients admitted with ventricular tachyarrhythmias. The following were the main findings of the study:

1. VF was shown to be the most common type of ventricular tachyarrhythmias in patients admitted with AMI and patients considered at lower risk for ventricular tachyarrhythmias, whereas s-VT was more frequently observed in patients with IHD.
2. In patients with AMI and in patients considered at lower risk for ventricular tachyarrhythmias, s-VT and VF were associated with comparable risk of all-cause mortality, whereas s-VT was associated with impaired prognosis compared to ns-VT.
3. Patients admitted with VF showed a higher risk of all-cause mortality compared to s-VT within IHD patients, whereas the risk of long-term mortality was comparable in s-VT and ns-VT.
4. The type of index arrhythmia had no impact on long-term mortality in patients with NICM.

Studies investigating the prevalence and prognostic impact of ventricular tachyarrhythmias are heterogenous and usually rely on pre-selected study populations [1,22]. Data are derived either from patient cohorts suffering from acute coronary syndrome, cardiogenic shock or cardiac arrest [23,24,25,26], or focus only on patients undergoing coronary angiography or implantation of an ICD [25,27,28,29]. There are also large reports based on nationwide statistical reports by health institutes, which focus more on broad disease groups, such as cardiac death, but do not allow further discrimination into underlying different types of ventricular tachyarrhythmias, exact comorbidities, or cardiac therapies and interventions [2,30], which represents a strength of the present study.

Ventricular tachyarrhythmias were reported to occur in approximately 8% of AMI patients [31]. However, despite earlier door-to-balloon times, an improved nationwide health-care supply and better revascularization strategies, the incidence of AMI-related ventricular arrhythmias has decreased [32,33]. AMI-related ventricular arrhythmias may result from zones of slow conduction and block leading to re-entry, or by focal triggers as a consequence of high sympathetic tone and mechanical stretching [34,35]; however, studies investigating the prognostic role of different types of ventricular tachyarrhythmias are scarce. The present study showed comparable risk of all-cause mortality in patients with s-VT and VF, along with an increased risk of the composite arrhythmic endpoint in s-VT, which may be related to an increased concomitant scar burden in patients with s-VT. Thus, specifically re-entry was shown to be a typical mechanism in patients with increased scar burden. Although only a minor part of the study population underwent cardiac magnet resonance tomography, we tried to adjust for the extent of scar burden by adjusting multivariable risk prediction models for the presence of prior AMI, CABG and CTO in patients with AMI and IHD.

Despite the risk of degeneration into VF, patients with VT and VF are often grouped together, although it was demonstrated that baseline characteristics of patients with VT and VF differ [36]. Thus, it was demonstrated that mean LVEF is typically lower in patients with VT [37,38], whereas higher rates of AMI were reported in VF patients [37]. These findings are in line with the present study, where increased rates of concomitant AMI were observed in VF patients and LVEF < 35% was most common in s-VT. However, studies focusing on ventricular tachyarrhythmias were predominantly published in the last century [37,38]. By now, the treatment of patients with cardiovascular disease has significantly improved, leading to increased rates of ICD implantation, higher supply with cardiovascular drugs and improved coronary revascularization strategies [39]. Along with this, patients’ characteristics have significantly changed, leading to an older study population with an increased proportion of multi-morbid patients. Therefore, European guidelines state the need to re-evaluate treatment strategies for patients at increased risk of SCD in the current medicine era [7]. In line with this, more than two out of three patients had an ICD in the s-VT group in the present study, whereas ICD implantation rate was lower in VF, presumably as a consequence of a higher rate of reversible causes, such as AMI in patients admitted with VF.

There are few studies available focusing on arrhythmia recurrences related to the type of index tachyarrhythmia. A sub-study of the AVID trial found increased risk for appropriate ICD therapies in patients with VT compared to VF at 31 months of follow-up [36]. In line with this, the present study found the highest risk of recurrent ventricular tachyarrhythmias in patients with s-VT in the subgroup of patients with AMI and NICM, which may be related to an increased arrhythmogenic substrate and scar-related re-entry, and a lower rate of reversible causes of ventricular tachyarrhythmias [10,40].

This study has several limitations. This observational and retrospective registry-based analysis reflects a realistic picture of consecutive health-care supply of high-risk patients presenting with ventricular tachyarrhythmias. The lost to follow-up rate regarding the evaluated endpoint of all-cause mortality was minimal. Patients not surviving out of hospital CPR and not being transferred to the heart center were not included in this study. The proportion of patients with slow VT and polymorphic VT was too small (<3%) to merit a separate sub-analysis. Due to the single-center design of the study, rehospitalization rates, recurrent ventricular tachyarrhythmias, as well as appropriate ICD therapies, were documented at one institution only. Only a minor part of the study population underwent cardiac magnet resonance imaging; therefore, no adjustment for scar burden was performed. In line with this, the presence of prior AMI, CABG and CTO were used as surrogate parameters for scar burden in the AMI and IHD group. Since heart transplantation is not performed at our institution, the number of patients undergoing heart transplantation during follow-up was not assessed for the present study.

In conclusion, the present study suggested that higher rates of AMI and out-of-hospital cardiac arrest were observed in patients with VF, whereas s-VT patients presented with higher rates of prior heart failure and lower LVEF on admission. At 2.5 years, VF was associated with the highest risk of all-cause mortality in patients with IHD, whereas s-VT and VF were associated with comparable prognosis in patients with AMI and in patients considered as the lower-risk group.

## Figures and Tables

**Table 1:** Baseline characteristics of the entire study cohort.

Characteristic	Ns-VT (n = 746)		*p* Value *	s-VT (n = 618)		*p* Value †	VF (n = 1058)		*p* Value ±
**Age**, median (range)	68 (15–95)		0.391	68 (15–97)		0.774	66 (14–92)		0.096
**Males**, n (%)	535	(72)	0.975	462	(75)	0.207	741	(70)	**0.038**
**Cardiovascular risk factors**, n (%)									
Arterial hypertension	461	(62)	**0.001**	370	(60)	0.468	533	(50)	**0.001**
Diabetes mellitus	195	(26)	0.973	166	(27)	0.764	271	(26)	0.575
Hyperlipidemia	222	(30)	0.064	187	(30)	0.841	251	(24)	**0.003**
Smoking	207	(28)	0.356	151	(24)	0.166	284	(27)	0.278
Cardiac family history	74	(10)	0.391	60	(10)	0.896	88	(8)	0.333
**Comorbidities at index**, n (%)									
Prior heart failure	184	(25)	0.172	198	(32)	**0.003**	173	(16)	**0.001**
Prior coronary artery disease	298	(40)	0.607	309	(50)	**0.001**	342	(32)	**0.001**
Prior myocardial infarction	165	(22)	0.763	201	(33)	**0.001**	179	(17)	**0.001**
Acute myocardial infarction	157	(21)	**0.001**	91	(15)	**0.003**	443	(42)	**0.001**
Non-ischemic cardiomyopathy	53	(7)	**0.040**	52	(8)	0.366	32	(3)	**0.001**
Channelopathy	21	(3)	0.151	15	(2)	0.656	52	(5)	**0.012**
Atrial fibrillation	235	(32)	0.172	216	(35)	0.178	266	(25)	**0.001**
Cardiogenic shock	38	(5)	**0.001**	83	(13)	**0.001**	282	(27)	**0.001**
CPR	59	(8)	**0.001**	160	(26)	**0.001**	924	(87)	**0.001**
Out of hospital	14	(2)	**0.001**	55	(9)	**0.001**	595	(56)	**0.001**
In hospital	45	(6)	**0.001**	105	(17)	**0.001**	329	(31)	**0.001**
Chronic kidney disease	288	(39)	**0.001**	313	(52)	**0.001**	593	(59)	**0.007**
COPD	72	(10)	0.213	65	(11)	0.596	71	(7)	**0.006**
**LVEF, n (%)**									
≥55%	200	(34)	0.060	98	(21)	**0.001**	237	(34)	**0.001**
54–45	88	(15)		47	(10)		109	(16)	
44–35%	105	(18)		103	(22)		141	(20)	
<35%	201	(34)		227	(48)		212	(30)	
Not documented	152	-		143	-		359	-	**-**
**Coronary angiography at index**, n (%)	432	(58)	0.573	311	(50)	**0.005**	680	(64)	**0.001**
No evidence of CAD	154	(36)	**0.001**	85	(27)	**0.003**	132	(19)	**0.006**
1-vessel disease	81	(19)		63	(29)		184	(27)	
2-vessel disease	106	(25)		63	(20)		167	(25)	
3-vessel disease	91	(21)		100	(32)		197	(29)	
CABG	61	(14)	0.348	60	(19)	0.060	62	(9)	**0.001**
Chronic total occlusion	76	(18)	0.071	76	(24)	**0.023**	140	(21)	0.173
PCI	149	(35)	**0.001**	87	(28)	0.060	410	(60)	**0.001**
**Electrical therapies at index, n (%)**									
Electrophysiological examination	231	(31)	**0.001**	250	(41)	**0.001**	113	(11)	**0.001**
VT ablation therapy	55	(7)	**0.004**	67	(11)	**0.025**	9	(0.9)	**0.001**
**Patients discharged**, n (%)	660	(88)	**0.001**	466	(75)	**0.001**	580	(55)	**0.001**
**Medication at discharge**, n (%)									
Beta-blocker	532	(81)	0.315	385	(83)	0.392	437	(75)	**0.004**
ACE-inhibitor	399	(61)	0.485	296	(64)	0.312	355	(61)	0.443
ARB	81	(13)	0.137	54	(12)	0.710	51	(9)	0.129
Aldosterone antagonist	71	(11)	0.875	64	(14)	0.130	46	(8)	**0.002**
Amiodarone	79	(12)	**0.003**	112	(24)	**0.001**	68	(12)	**0.001**
**Presence of an ICD**, n (%)	254	(39)	**0.001**	312	(67)	**0.001**	271	(47)	**0.001**

ACE, angiotensin converting enzyme; ARB, angiotensin II receptor blocker; CABG, coronary artery bypass grafting; CAD, coronary artery disease; COPD, chronic obstructive pulmonary disease; CPR, cardiopulmonary resuscitation; LVEF, left ventricular ejection fraction; ICD, implantable cardioverter-defibrillator; ns-VT, non-sustained ventricular tachycardia; PCI, percutaneous coronary intervention; s-VT, sustained VT; VT, ventricular fibrillation. * comparison of ns-VT versus s-VT plus VF. † comparison of s-VT versus ns-VT. ± comparison of VF versus s-VT. Level of significance *p* < 0.05. Bold type indicates statistical significance.

## Data Availability

The datasets used and/or analyzed during the current study are available from the corresponding author on reasonable request.

## References

[1] Zeppenfeld K., Tfelt-Hansen J., de Riva M., Winkel B.G., Behr E.R., Blom N.A., Charron P., Corrado D., Dagres N., de Chillou C. (2022). 2022 ESC Guidelines for the management of patients with ventricular arrhythmias and the prevention of sudden cardiac death. Eur. Heart J..

[2] Zheng Z.-J., Croft J.B., Giles W.H., Mensah G. (2001). Sudden cardiac death in the United States, 1989 to 1998. Circulation.

[3] Shuvy M., Qiu F., Lau G., Koh M., Dorian P., Geri G., Lin S., Ko D.T. (2019). Temporal trends in sudden cardiac death in Ontario, Canada. Resuscitation.

[4] Wong C.X., Brown A., Lau D.H., Chugh S.S., Albert C.M., Kalman J.M., Sanders P. (2019). Epidemiology of sudden cardiac death: Global and regional perspectives. Heart Lung Circ..

[5] Everett B.M., Moorthy M.V., Tikkanen J.T., Cook N.R., Albert C.M. (2020). Markers of myocardial stress, myocardial injury, and sub-clinical inflammation and the risk of sudden death. Circulation.

[6] Markwerth P., Bajanowski T., Tzimas I., Dettmeyer R. (2021). Sudden cardiac death-update. Int. J. Legal Med..

[7] Priori S.G., Blomström-Lundqvist C., Mazzanti A., Blom N., Borggrefe M., Camm J., Elliott P.M., Fitzsimons D., Hatala R., Hindricks G. (2015). 2015 ESC guidelines for the management of patients with ventricular arrhythmias and the prevention of sudden cardiac death: The task force for the management of patients with ventricular arrhythmias and the prevention of sudden cardiac death of the european society of cardiology (ESC). Endorsed by: Association for European Paediatric and Congenital Cardiology (AEPC). Eur. Heart J..

[8] Enriquez A., Frankel D.S., Baranchuk A. (2017). Pathophysiology of ventricular tachyarrhythmias: From automaticity to reentry. Herzschrittmacherther. Elektrophysiol..

[9] Issa Z.F., Miller J.M., Zipes D.P., Issa Z.F., Miller J.M., Zipes D.P. (2019). 3—Electrophysiological mechanisms of cardiac arrhythmias. Clinical Arrhythmology and Electrophysiology.

[10] Zipes D. (2003). Mechanisms of clinical arrhythmias. J. Cardiovasc. Electrophysiol..

[11] Jung R.G., Di Santo P., Mathew R., Simard T., Parlow S., Weng W., Abdel-Razek O., Malhotra N., Cheung M., Hutson J.H. (2022). Arrhythmic events and mortality in patients with cardiogenic shock on inotropic support: Results of the DOREMI randomized trial. Can. J. Cardiol..

[12] Zeppenfeld K., Wijnmaalen A.P., Ebert M., Baldinger S.H., Berruezo A., Catto V., Vaseghi M., Arya A., Kumar S., de Riva M. (2022). Clinical outcomes in patients with dilated cardiomyopathy and ventricular tachycardia. J. Am. Coll. Cardiol..

[13] Weizman O., Marijon E., Narayanan K., Boveda S., Defaye P., Martins R., Deharo J., Laurent G., Klug D., Sadoul N. (2022). Incidence, characteristics, and outcomes of ventricular fibrillation complicating acute myocardial infarction in women admitted alive in the hospital. J. Am. Heart Assoc..

[14] Behnes M., Mashayekhi K., Weiß C., Nienaber C., Lang S., Reiser L., Bollow A., Taton G., Reichelt T., Ellguth D. (2018). Prognostic impact of acute myocardial infarction in patients presenting with ventricular tachyarrhythmias and aborted cardiac arrest. J. Am. Heart Assoc..

[15] Rusnak J., Behnes M., Weiß C., Nienaber C., Reiser L., Schupp T., Bollow A., Taton G., Reichelt T., Ellguth D. (2020). Non-ischemic compared to ischemic cardiomyopathy is associated with increasing recurrent ventricular tachyarrhythmias and ICD-related therapies. J. Electrocardiol..

[16] Weidner K., Behnes M., Weiß C., Nienaber C., Schupp T., Reiser L., Bollow A., Taton G., Reichelt T., Ellguth D. (2019). Increasing age is associated with recurrent ventricular tachyarrhythmias and appropriate ICD therapies secondary to documented index ventricular tachyarrhythmias. Eur. Geriatr. Med..

[17] Weidner K., Behnes M., Schupp T., Rusnak J., Reiser L., Taton G., Reichelt T., Ellguth D., Engelke N., Bollow A. (2019). Prognostic impact of chronic kidney disease and renal replacement therapy in ventricular tachyarrhythmias and aborted cardiac arrest. Clin. Res. Cardiol..

[18] Behnes M., Mashayekhi K., Kuche P., Kim S.-H., Schupp T., Von Zworowsky M., Reiser L., Bollow A., Taton G., Reichelt T. (2021). Prognostic impact of coronary chronic total occlusion on recurrences of ventricular tachyarrhythmias and ICD therapies. Clin. Res. Cardiol..

[19] Rusnak J., Behnes M., Schupp T., Reiser L., Bollow A., Taton G., Reichelt T., Ellguth D., Engelke N., Hoppner J. (2018). COPD increases cardiac mortality in patients presenting with ventricular tachyarrhythmias and aborted cardiac arrest. Respir. Med..

[20] Weidner K., Behnes M., Schupp T., Rusnak J., Reiser L., Bollow A., Taton G., Reichelt T., Ellguth D., Engelke N. (2018). Type 2 diabetes is independently associated with all-cause mortality secondary to ventricular tachyarrhythmias. Cardiovasc. Diabetol..

[21] Rusnak J., Behnes M., Weiß C., Nienaber C., Reiser L., Schupp T., Bollow A., Taton G., Reichelt T., Ellguth D. (2020). Impact of left ventricular ejection fraction on recurrent ventricular tachyarrhythmias in recipients of implantable cardioverter defibrillators. Cardiology.

[22] Zipes D.P., Camm A.J., Borggrefe M., Buxton A.E., Chaitman B., Fromer M., Gregoratos G., Klein G., Moss A.J., Myerburg R.J. (2006). ACC/AHA/ESC 2006 guidelines for management of patients with ventricular arrhythmias and the prevention of sudden cardiac death: A report of the american college of cardiology/american heart association task force and the european society of cardiology committee for practice guidelines (writing committee to develop guidelines for management of patients with ventricular arrhythmias and the prevention of sudden cardiac death): Developed in collaboration with the European heart rhythm association and the heart rhythm society. Circulation.

[23] Gorjup V., Radsel P., Kocjancic S.T., Erzen D., Noc M. (2007). Acute ST-elevation myocardial infarction after successful cardiopulmo-nary resuscitation. Resuscitation.

[24] Kern K.B., Lotun K., Patel N., Mooney M.R., Hollenbeck R.D., McPherson J.A., McMullan P.W., Unger B., Hsu C.H., Seder D.B. (2015). Outcomes of comatose cardiac arrest survivors with and without st-segment elevation myocardial infarction: Importance of coronary angiography. JACC Cardiovasc. Interv..

[25] Patel N., Patel N.J., Macon C.J., Thakkar B., Desai M., Rengifo-Moreno P., Alfonso C.E., Myerburg R.J., Bhatt D.L., Cohen M. (2016). Trends and outcomes of coronary angiography and percutaneous coronary intervention after out-of-hospital cardiac arrest associated with ventricular fibrillation or pulseless ventricular tachycardia. JAMA Cardiol..

[26] Mylotte D., Morice M.-C., Eltchaninoff H., Garot J., Louvard Y., Lefèvre T., Garot P. (2013). Primary percutaneous coronary intervention in patients with acute myocardial infarction, resuscitated cardiac arrest, and cardiogenic shock: The role of primary multivessel revascularization. JACC Cardiovasc. Interv..

[27] Sedlacek K., Ruwald A.C., Kutyifa V., McNitt S., Thomsen P.E., Klein H., Stockburger M., Wichterle D., Merkely B., Swissa M. (2015). The effect of ICD programming on inappropriate and appropriate ICD therapies in ischemic and nonischemic cardiomyopathy: The MADIT-RIT trial. J. Cardiovasc. Electrophysiol..

[28] Suleiman M., Goldenberg I., Samniah N., Rosso R., Marai I., Pekar A., Khalameizer V., Militianu A., Glikson M. (2015). Outcome of patients with advanced heart failure who receive device-based therapy for primary prevention of sudden cardiac death: Insights from the Israeli ICD registry. Pacing Clin. Electrophysiol..

[29] Sun W.P., Li C.L., Guo J.C., Zhang L.X., Liu R., Zhang H.B., Zhang L. (2016). Long-term efficacy of implantable cardiac resynchronization therapy plus defibrillator for primary prevention of sudden cardiac death in patients with mild heart failure: An updated meta-analysis. Heart Fail. Rev..

[30] Nichol G., Guffey D., Stiell I.G., Leroux B., Cheskes S., Idris A., Kudenchuk P.J., Macphee R.S., Wittwer L., Rittenberger J.C. (2015). Post-discharge outcomes after resuscitation from out-of-hospital cardiac arrest: A roc primed substudy. Resuscitation.

[31] Pan J., Zhang Q., Lei L., Chen Y., Li G., Liang H., Lu J., Zhang X., Tang Y., Pu J. (2022). Impact of the CAFFR-guided functional syntax score on ventricular tachycardia/fibrillation development in patients with acute myocardial infarction. Front. Cardiovasc. Med..

[32] Latini R., Maggioni A.P., Flather M., Sleight P., Tognoni G. (1995). ACE inhibitor use in patients with myocardial infarction. Summary of evidence from clinical trials. Circulation.

[33] Volpi A., De Vita C., Franzosi M.G., Geraci E., Maggioni A.P., Mauri F., Negri E., Santoro E., Tavazzi L., Tognoni G. (1993). Determinants of 6-month mortality in survivors of myocardial infarction after thrombolysis. Results of the GISSI-2 data base. The Ad hoc working group of the gruppo italiano per lo studio della sopravvivenza nell’Infarto miocardico (GISSI)-2 data base. Circulation.

[34] Wit A., Rosen M. (1986). The Heart and Cardiovascular System.

[35] Bhar-Amato J., Davies W., Agarwal S., Trust P.E.P.H.N.F. (2017). Ventricular arrhythmia after acute myocardial infarction: ‘The perfect storm’. Arrhythmia Electrophysiol. Rev..

[36] Raitt M.H., Klein R.C., Wyse D., Wilkoff B.L., Beckman K., Epstein A.E., Coromilas J., Friedman P.L., Martins J., Ledingham R.B. (2003). Comparison of arrhythmia recurrence in patients presenting with ventricular fibrillation versus ventricular tachycardia in the antiarrhythmics versus implantable defibrillators (AVID) trial. Am. J. Cardiol..

[37] Adhar G.C., Larson L.W., Bardy G.H., Greene H.L. (1988). Sustained ventricadoular arrhythmias: Differences between survivors of car-diac arrest and patients with recurrent sustained ventricular tachycardia. J. Am. Coll. Cardiol..

[38] Raitt M.H., Dolack G.L., Kudenchuk P.J., Poole J.E., Bardy G.H. (1995). Ventricular arrhythmias detected after transvenous defibrillator implantation in patients with a clinical history of only ventricular fibrillation: Implications for use of implantable defibrillator. Circulation.

[39] Maggioni A.P., Anker S.D., Dahlström U., Filippatos G., Ponikowski P., Zannad F., Amir O., Chioncel O., Leiro M.C., Drozdz J. (2013). Are hospitalized or ambulatory patients with heart failure treated in accordance with European society of cardiology guidelines? Evidence from 12,440 patients of the ESC heart failure long-term registry. Eur. J. Heart Fail..

[40] Ajijola O.A., Tung R., Shivkumar K. (2014). Ventricular tachycardia in ischemic heart disease substrates. Indian Heart J..

